# Enhanced inhibitory effect of curcumin via reactive oxygen species generation in human nasopharyngeal carcinoma cells following purple-light irradiation

**DOI:** 10.3892/ol.2013.1341

**Published:** 2013-05-08

**Authors:** DUJUAN WANG, JIANG HU, LIN LV, XIUWEN XIA, JIANZHONG LIU, XIAOYUAN LI

**Affiliations:** 1 Departments of Pathology and Pathophysiology, Zhongshan School of Medicine, Sun Yat-Sen University, Guangzhou, Guangdong 510080, P.R. China; 2 Biomedical Engineering, Zhongshan School of Medicine, Sun Yat-Sen University, Guangzhou, Guangdong 510080, P.R. China; 3 Cell Biology, Zhongshan School of Medicine, Sun Yat-Sen University, Guangzhou, Guangdong 510080, P.R. China

**Keywords:** apoptosis, cell cycle arrest, reactive oxygen species

## Abstract

Curcumin, a traditional medicine, exhibits anti-carcinogenic properties in various cell lines and animals. As a phenolic compound, curcumin is light-sensitive and photoactived curcumin exhibits a greater anticancer effect compared with curcumin alone. However, the mechanisms by which curcumin inhibits tumor cell growth in human nasopharyngeal carcinoma (NPC) cells following purple light (PL) irradiation remains unclear. In the present study, CNE1 and CNE2 cells were treated with curcumin and exposed to PL at various energy densities to determine the anticancer activity of curcumin using MTT assays, staining and flow cytometry. The subsequent changes in the cell viability, morphology, cell cycle, apoptosis and reactive oxygen species (ROS) generation were measured. Curcumin inhibited cell growth in a dose-dependent manner. CNE1 and CNE2 cells tended to be arrested at the S or G_2_/M cell cycle stages following curcumin treatment and the levels of ROS increased in a time-dependent manner. However, after treatment with curcumin followed by PL irradiation, the levels of cytotoxicity and apoptotic cell death were significantly increased compared with the curcumin-only group. ROS generation was also enhanced in an energy-dependent manner. In summary, following PL irradiation, the anti-cancer effect of curcumin in human NPC cells was increased through apoptosis and cell cycle arrest.

## Introduction

Human nasopharyngeal carcinoma (NPC) is one of the most common types of cancer in Southern China (1). NPC is a relatively radiosensitive disease, although the majority of NPC patients suffer from recurrence and metastasis within 1.5 years of treatment (2). Chemotherapy is a necessary treatment for NPC patients (3) and the potential complications and side-effects (e.g., neutropenia and immunosuppression) of these drugs limit the application of chemotherapy in NPC. Thus the identification of novel anticarcinogenic agents with high efficacy, low toxicity and known mechanisms of action is crucial. A number of studies have focused on extracting active ingredients from natural plants to prevent and treat cancer and investigating their anticancer mechanisms (4).

Curcumin, a phenolic compound extracted from the plant *Curcuma longa*, exhibits a wide range of pharmacological effects, including anti-inflammatory, anticarcinogenic, hypocholesterolemic and anti-infection activities. Due to its regulation of multiple cellular pathways, studies have focused on its clinical importance for the treatment of different types of cancer (5). More significantly, curcumin has no or low cytotoxicity in normal cells *in vitro* (6) and inhibited carcinogenesis of various types of cancer without notable treatment-related toxicity in a phase I study (7). Curcumin is safe in humans; a dose of 10 g/day has been shown not to produce treatment-related toxicity (8).

Curcumin exhibits photobiological and photosensitizing activity (9). It has been reported that curcumin combined with light irradiation exhibits more marked anticancer effects than curcumin without irradiation (10). Certain studies have used curcumin as a photosensitizer in photodynamic therapy to treat cancer (11,12). Curcumin is sensitive to ultraviolet and visible light (13). The greatest absorption peak of curcumin is at 408 nm (14), so in the present study a purple LED light (405 nm) was used to excite curcumin. Thus far, the direct cytotoxic effect of curcumin on NPC cells following purple-light (PL) irradiation has not been reported and this was the main purpose of the present study.

## Materials and methods

### Chemicals and reagents

Curcumin, 3-(4,5-dimethyl-2-thiazolyl)-2,5-diphenyl-2H-tetrazolium bromide (MTT) and propidium iodide (PI) were obtained from Sigma-Aldrich Chemical Co. (St. Louis, MO, USA). 2,7-Dichlorodihydrofluorescein diacetate (DCFH-DA) and Hoechast 33342 were purchased from Molecular Probes (Invitrogen, Eugene, OR, USA). The culture medium RPMI-1640, fetal bovine serum (FBS), penicillin-streptomycin and L-glutamine were purchased from GIBCO BRL (Invitrogen, Grand Island, NY, USA).

### Cell culture

The human NPC cell lines, CNE1 and CNE2, were obtained from the Cancer Center of Sun Yat-Sen University (Guangzhou, China) and cultured in RPMI-1640 medium containing 10% FBS and penicillin-streptomycin sulfate. All cell lines were incubated at 37°C in an atmosphere of 5% CO_2_.

### Cell viability assays

The MTT assay was used to evaluate the anticancer effect on cell viability. For the curcumin group, the cells were seeded at a density of 1×10^4^/well into 96-well plates for 24 h and incubated with curcumin for 2 h. Fresh medium was then added into each well. The curcumin followed by PL irradiation groups were then exposed to PL irradiation at various energy densities and fresh medium was added. After incubation for 24 h, MTT reagent was added and the cells were incubated for 4 h, lysed with DMSO and quantitated using a plate reader.

### Morphological changes

The cells were plated on to 6-well plates at a density of 2×10^5^ cells/well overnight and then divided into three groups (control, curcumin and curcumin + PL groups). After 24 h, the cells were fixed with methanol and then stained with Hoechst 33342 (10 *μ*g/ml for 15 min) and washed with PBS. A fluorescence microscope was used to observe the apoptotic morphological changes.

### Cell cycle and apoptosis determination

CNE1 and CNE2 cells (∼3×10^5^ cells/well) in 6-well plates were incubated with 40 *μ*M curcumin for 2 h and then irradiated with PL at 0.2 J/cm^2^. The cells were harvested by centrifugation and fixed in cold 70% ethanol at 4°C overnight (≥12 h). The fixed cells were washed with PBS and stained with PI containing RNase A at 10 *μ*g/ml. The cells were separated by flow cytometry (FACScalibur, Becton Dickinson, San Jose, CA, USA) and the results were analyzed using ModFit Software. The sub-G_1_ groups (apoptosis) were calculated and analyzed using CellQuest (Becton-Dickinson) and ModFit Software (Verity Software House Inc., Topsham, ME, USA).

### Detection of reactive oxygen species (ROS)

CNE1 and CNE2 cells (∼3×10^5^ cells/well) were seeded into 6-well plates overnight and treated using various methods. The cells were harvested and washed twice, re-suspended in 500 *μ*l of DCFH-DA (10 *μ*M) and the levels of ROS were analyzed by flow cytometry.

### Statistical analysis

At least three independent experiments were performed for the statistical evaluation. Data are presented as the mean ± SEM. The statistical analysis of the results was performed using the Student’s t-test (two-tailed, unpaired) if two groups were compared or one-way analysis of variance if there were more than two groups. P<0.05 was considered to indicate statistically significant differences.

## Results

### Enhanced cytotoxicity of curcumin in NPC cells following PL irradiation

**Figure 1.**
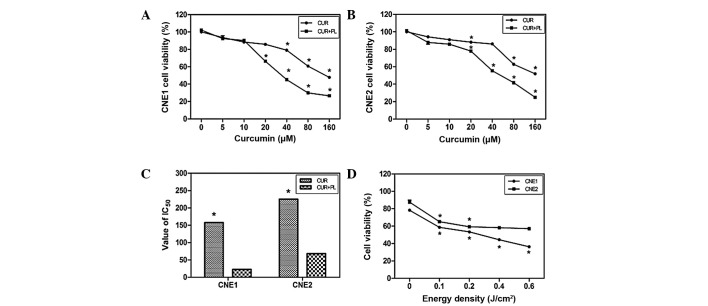
Viability of CNE1 and CNE2 cells following various treatments. (A and B) In the curcumin group, the CNE1 and CNE2 cells were treated with curcumin (5, 10, 20, 40, 80 and 160 *μ*M) for 2 h, then washed with fresh medium and after 24 h the percentage of living cells was determined. In the curcumin + PL group, the cells were incubated with curcumin for 2 h, and washed, followed by PL irradiation at 0.2 J/cm^2^. The percentage of living cells in the treated groups vs. the untreated controls was then measured. (C) IC_50_ of NPC cells in the curcumin and curcumin + PL groups. (D) NPC cells were incubated with curcumin (40 *μ*M) for 2 h, washed, then irradiated with PL at various energy densities (0.1, 0.2, 0.4 and 0.6 J/cm^2^) and subsequently the cell viability was calculated. Data are the mean ± SE; ^*^P<0.01 vs. control. PL, purple light; NPC, nasopharyngeal carcinaoma; CUR, curcumin.

As shown in Fig. 1, the percentage of viable cells in the curcumin groups decreased with the IC_50_ at 157.5 *μ*M in the CNE1 cells and 225.2 *μ*M in the CNE2 cells. Curcumin treatment followed by PL irradiation enhanced the effect in an energy density-dependent manner (Fig. 1D). The IC_50_ values of the CNE1 and CNE2 cells treated with curcumin and PL irradiation at 0.2 J/cm^2^ decreased to 22.52 and 68.2 *μ*M, respectively. Treatment with 40 *μ*M curcumin and 0.2 J/cm^2^ energy density was used in the subsequent experiments.

### Effect of curcumin on NPC cell morphology following PL irradiation

**Figure 2.**
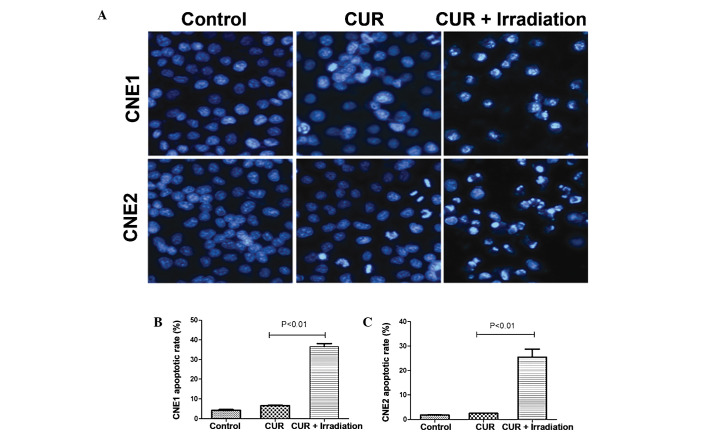
Apoptotic cell death enhanced by curcumin following PL irradiation. CNE1 and CNE2 cells were incubated with curcumin (40 *μ*M) for 2 h, washed and then irradiated with PL at 0.2 J/cm^2^ for 24 h. (A) CNE1 and CNE2 cells were stained with 10 *μ*g/ml Hoechst 33342 for 15 min to assess the apoptotic cell changes under a fluorescence microscope (×40). (B and C) Percentages of apoptotic cells stained with PI were measured by flow cytometry. Results are representative of three experiments. Data are the mean ± SE. PL, purple light; PI, propidium iodide; CUR, curcumin.

Alterations to the cells’ nuclear morphology were studied using Hoechst 33342 staining to assess whether curcumin followed by PL irradiation induced NPC cell death by apoptosis. As shown in Fig. 2A, the typical morphological features of apoptosis were observed, as characterized by marked chromatin condensation and nuclear fragmentation. The number of cells exhibiting nuclei with condensed chromatin increased significantly after treatment with curcumin followed by PL irradiation.

### Cell cycle arrest and apoptosis of NPC cells after treatment with curcumin followed by PL irradiation

The sub-G_1_ peaks indicating the proportion of apoptotic cells increased to 36.6% in the CNE1 cells and 25.5% in the CNE2 cells when curcumin (40 *μ*M) was exposed to 0.2 J/cm^2^ PL irradiation compared with the curcumin treatment group (Fig. 2B and C).

**Figure 3.**
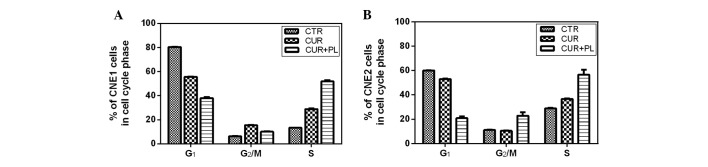
Cell cycle population of CNE1 and CNE2 cells was determined following various treatments for 24 h. CNE1 and CNE2 cells were treated with curcumin (40 *μ*M) for 2 h, washed and exposed by PL irradiation at 0.2 J/cm^2^. Cells were harvested and the cell cycle was analyzed. Data are the mean ± SE of three experiments. PL, purple light; CTR, control; CUR, curcumin.

The cell cycle distribution of the CNE1 and CNE2 cells after treatment with curcumin and curcumin followed by PL irradiation for 24 h is shown in Fig. 3. The majority of CNE1 cells treated with curcumin (40 *μ*M) followed by PL irradiation at 0.2 J/cm^2^ were arrested at the S phase and the proportion of S phase cells increased to 51.9%. The proportion of G_2_/M phase cells among the CNE2 cells was double that of the control group treated with curcumin and the proportion of S phase cells was 56.6%.

### Effect of curcumin on ROS production in NPC cells following PL irradiation

**Figure 4.**
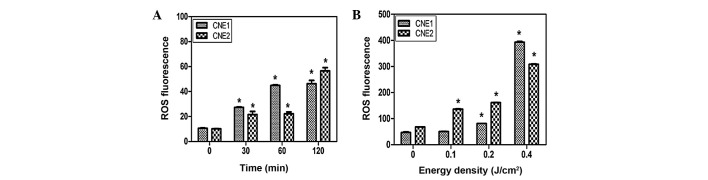
ROS generation and cell viability in CNE1 and CNE2 cells following various treatments. (A) Curcumin (40 *μ*M) was added to CNE1 and CNE2 cells which were incubated for 0.5-2 h. The cells were then stained with 10 *μ*M DCFH-DA for 30 min. The levels of intracellular ROS were estimated using flow cytometry. (B) CNE1 and CNE2 cells were incubated with curcumin (40 *μ*M) for 2 h, washed and exposed to PL at various energy densities (0.1, 0.2 and 0.4 J/cm^2^). The ROS levels were determined immediately. Data are the mean ± SE of three experiments. ^*^P<0.01 vs. control. ROS, reactive oxygen species; DCFH-DA, 2,7-dichlorodihydrofluorescein diacetate; PL, purple light.

CNE1 and CNE2 cells were incubated with curcumin (40 *μ*M) for 30, 60 and 120 min. The relative level of ROS increased from 10.5 to 46.2 in CNE1 cells and 10.1 to 56.5 in CNE2 cells (Fig. 4A) between 0 and 120 min. Furthermore, the ROS fluorescence value of the CNE1 cells increased from 9.6 to 392.8 between 0 and 0.4 J/cm^2^, while in CNE2 cells it increased to 308.1. Compared with the curcumin group, ROS generation was greatly increased when the cells were incubated with curcumin for 2 h followed by PL irradiation at an energy density of 0.2 J/cm^2^.

## Discussion

Curcumin, which possesses anticancer activity, is widely used as a chemopreventive agent in numerous types of cancer, including breast, lung, colon, prostate, stomach, kidney, ovary, brain and blood cancer (15). Few studies have focused on NPC (16,17). In China, >95% of NPCs are nonkeratinizing carcinoma while <5% are keratinizing carcinoma, and thus CNE1 (keratinizing carcinoma) and CNE2 (nonkeratinizing carcinoma) cells were used to represent the two main histological types in the present study (18). As reported previously, curcumin is sensitive to sun- or UV light (19). When curcumin was combined with exposure to visible (20) or blue-filtered light (11), it exhibited more marked anticancer effects than by itself. In addition, it was previously reported that the photobiological activity of curcumin was due to its excited state rather than the products of the photodegradation of curcumin, such as ferulic acid and vanillin (21). Koon *et al* also clarified that curcumin was rapidly absorbed in the first 1 h. Due to this, the NPC cells were incubated with curcumin for 2 h, washed with fresh medium and finally exposed to PL to produce the excited state of curcumin (11).

In the present study, it was observed that curcumin was cytotoxic towards CNE1 and CNE2 cells in a dose-dependent manner and the cytotoxicity in CNE1 cells was more marked than that in CNE2 cells. The cytotoxic effect of curcumin following PL irradiation was greater than that of curcumin alone. Curcumin treatment followed by PL irradiation enhanced the effect in an energy density-dependent manner and exhibited increased cytotoxicity compared with the curcumin group.

The most studied property of photo-actived curcumin is its pro-apoptotic effect. Park and Lee observed the photosensitizer effect of curcumin in UVB-irradiated HaCaT cells via the activation of caspase pathways (12). Dujic *et al* demonstrated the effect on apoptosis, showing the enhanced activation of caspase-9 (22). By contrast, Chan and Wu reported that curcumin inhibited apoptosis in photosensitized A431 cells (23). Thus curcumin had a two-sided effect which was dependent on the concentration, cell lines and cellular micro-environment. The present data demonstrated that curcumin treatment followed by PL irradiation induced apoptosis in NPC cells and the apoptotic effect was more marked than that of curcumin alone.

Curcumin passes through the plasma membrane and induce ROS generation. Intracellular ROS damage mitochondrial and nuclear DNA and lead to apoptosis (24). The photoexcited state of curcumin is able to increase the level of singlet-oxygen (14). Atsumi T also demonstrated that visible light irradiation following curcumin treatment greatly enhanced the pro-apoptotic effect due to the increase in ROS levels. The ROS levels were measured to demonstrate the important role of ROS in curcumin treatment followed by PL irradiation-induced apoptosis in NPC cells. From the present data, we suggest that ROS may be more important in photoactivated curcumin-induced apoptosis compared with curcumin alone.

Besides apoptosis, the dysregulation of the cell cycle also contributes to tumorigenesis (25). CNE1 and CNE2 cells were arrested at the S and G_2_/M phases as reported in breast cancer by Mehta *et al* (26). Furthermore, CNE1 cells treated with curcumin followed by PL irradiation were mainly arrested at the S phase, while CNE2 cells were arrested at the G_2_/M and S phases. Apoptotic induction and cell cycle arrest contribute to the anticancer effect of curcumin following PL irradiation.

In summary, curcumin treatment followed by PL irradiation enhances the cytotoxicity against CNE1 and CNE2 cells through the potential induction of apoptosis and ROS generation. The treatment promoted S or G_2_/M phase arrest in the two cell lines. Taken together, the data indicate that curcumin PL exposure may be a potentially effective therapy for NPC.
